# Stool-Based Pathogen Detection Offers Advantages as an Outcome Measure for Water, Sanitation, and Hygiene Trials

**DOI:** 10.4269/ajtmh.19-0639

**Published:** 2019-11-04

**Authors:** Joe Brown, Oliver Cumming

**Affiliations:** 1 School of Civil and Environmental Engineering, Georgia Institute of Technology, Atlanta, Georgia;; 2 Department of Disease Control, Faculty of Infectious and Tropical Diseases, London School of Hygiene and Tropical Medicine, London, United Kingdom

## Abstract

Most health impact trials of water, sanitation, and hygiene use caregiver-reported diarrhea in children as the primary outcome; this measure is known to be subject to considerable bias, especially when used in unblinded trials. Detection of enteric pathogens in stool or fecal waste via multiplex molecular methods may offer advantages over—and is complementary to—caregiver-reported diarrhea because these measures are objective, on the causal pathway from exposures of interest to disease outcomes, and increasingly feasible in high-burden countries.

Water, sanitation, and hygiene (WASH) health impact trials have typically used caregiver-reported childhood diarrhea as a primary outcome. This is justified in public health terms as childhood diarrhea is assumed to account for most of the WASH-attributable disease burden.^1^ As a primary outcome for health impact trials, however, diarrhea is problematic as it generally relies on self-reported information, with significant potential for bias, especially when used in unblinded trials.^2^ Beyond these long-standing concerns, important new studies have demonstrated that the etiology of childhood diarrhea in high-burden settings is diverse and varies significantly by setting, population, and age^3^; moreover, prevalence and detection of a range of enteric pathogen targets is very high in at-risk populations, even in apparently asymptomatic individuals.^4^ Here, we consider stool-based enteric pathogen detection as an alternative, novel outcome for WASH trials, offering several advantages over caregiver-reported diarrhea symptomology.

First, detection of enteric pathogens in stool is an objective outcome measurable in either quantal or quantitative multiplex assays and may include viruses, bacteria, protozoa, and helminths of interest. Such methods are supported by standard, validated procedures used in diagnostic assays, based on well-characterized gene targets, offering low detection limits and potential for quantification.^5,6^ A range of commercially available kits and custom assays^6–8^ have enabled simultaneous detection of multiple enteric pathogens via molecular methods, many supported by systematic studies reporting sensitivity and specificity, compared with alternative methods in different populations. These are powerful tools widely available for use globally; the required molecular facilities are available in most countries, including those with a high burden of diarrheal disease.

Second, stool-based enteric pathogen detection can identify etiologies of potential importance in populations of interest; such information may assist with developing more effective interventions to reduce exposure. Even in sites where information on the diarrheal disease burden is available, knowledge of potential underlying etiologies of enteric infection and disease remains critically important for the design of interventions to interrupt transmission. Dominant transmission pathways vary by setting and also by pathogen; effective interventions to control rotavirus infection in the United States will differ from those for cryptosporidiosis in Kenya.

Third, for an individual, detection of an enteric pathogen in stool is an unambiguous indication of past exposure to that pathogen. Regardless of symptomology and without indicating colonization or potential for current or future effects on gut health, the presence of a pathogen in stool is possible only if the individual has been exposed at some point before the specimen was collected. As WASH interventions are intended to reduce exposure to enteric pathogens, the use of straightforward exposure measures as proximal trial outcomes is justifiable. Enteric pathogen presence in the gut is a necessary precondition of enteric infection, itself on the causal pathway from primary WASH-related or fecal–oral exposure to WASH-related disease outcomes, notably diarrheal diseases,^3^ and various hypothesized sequelae, including environmental enteric dysfunction,^9^ adverse growth^10^ and cognitive impairment,^11^ impacts on the immune system,^12^ and poor oral vaccine performance.^13^

Despite some clear advantages, there are important limitations to stool-based enteric pathogen detection as an outcome measure in trials. The science in this area is evolving rapidly, and our understanding of the many underlying biological mechanisms is incomplete, particularly with respect to the health significance to an individual—if any—of shedding pathogens in stool when no symptoms are present.^8^ Pathogen detection in stool may not necessarily indicate an active infection and may not have clear implications for gut health. Although subclinical carriage has been found to be generally high in at-risk populations,^3,4^ enteric pathogens may act more as commensal members of the gut microbiota under some conditions. Enteric pathogen detection may also be inconsistent or ephemeral in stool based on standard diagnostic assays, and persistence of infections may vary according to a range of factors. Enteric pathogen shedding may not be very sensitive to changes in exposure over the relatively short time scales of impact trials; however, more work is needed on asymptomatic shedding of important enteric pathogens in longitudinal cohort studies to provide more evidence on this point. Most methods now under consideration for multiplex pathogen detection in stool are library-dependent, and therefore, detection will be limited to specific targets sought, potentially missing clinically relevant pathogens. Commercially available platforms for identification of enteric pathogens in stool may also lack essential transparency in methods, including full reporting of primers and probes that would allow for direct comparison across methods. Metagenomics may offer greater scope for detection of multiple targets, but will generally have a substantially higher limit of detection, lowering sensitivity for individual pathogens of interest, and may be less available in settings where risk is highest. Finally, bulk stool or rectal swab samples may be logistically difficult and, therefore, costly to obtain in large trials, limiting the use of such measures in practice. Using aggregate fecal waste streams, such as fecal sludges or wastewaters,^14,15^ may offer logistical advantages over collection of fecal specimens from individuals; however, this strategy is accompanied by other complications such as pathogen proliferation (for bacteria) and die-off, potentially masking important differences in shedding among individuals, and nonhuman sources. Wastewater monitoring is a common surveillance method supporting, for example, poliovirus eradication programs, but may also be more widely applied among other enteric targets. Whether pathogen detection in environmental matrices can provide a useful proxy outcome measure in trials remains to be seen; more research is required to understand whether and to what extent such signals may be sensitive to changes in disease prevalence or incidence at various scales.

Stool-based enteric pathogen detection offers several advantages over the conventional WASH trial outcome of caregiver-reported diarrhea. Ultimately, without more objective outcomes relating to specific diarrheal etiologies, rather than generalized and subjective symptoms, it will remain challenging to mobilize the most appropriate interventions, whether these be environmental, such as WASH, or medical, such as vaccination.
